# Medical News and Miscellany

**Published:** 1890-04

**Authors:** 


### Medical News and Miscellany

Dr. J. T. HARRINGTON has removed from El Paso to Abilene.

Dr. T. J. MILNER, ot Greenville, left on the 10th inst. for New York, to take a course at the Polyclinic. We regret that he will not be with us at Fort Worth; we will miss him.

JAS. KENNEDY, a well-known drnggist of San Antonio, and President of the State Pharmacy Association, graduated M. D. at the University of the City of New York recently. Will locate at San Antonio.

CRIMINAL LIBEL.—We are informed by readers of the Texas Health Journal that the editor of that publication, Dr. J. R. Briggs, is being prosecuted for criminal libel, in the courts at Dallas, by a Doctor Reeves, and that he makes a touching appeal to delinquent subscribers to pay up to enable him to defend the suit.

MARRIED.—On Thursday, March 6, at the residence of John A. Davis, Dr. D. B. McGee, of May, Brown co., Texas, to Miss Cora Diggs, of Grapeland, Houston county, Texas. The Doctor and his fair bride arrived in this city on Saturday night's train and left for the romantic city of May Sunday morning. Mrs. McGee is to be congratulated in capturing the Doctor, for many there be that have tried before but their efforts were futile. Mrs. McGee is an accomplished young lady of about sixteen sweet summers, and will add greatly to the circles of May society. The Doctor says she is his joy and pride in prosperity, his support and comforter in affliction. We wish this newly made couple a long, happy and prosperous life.—Ex.

WITHIN a very few days after this issue of the JOURNAL reaches its readers the Texas State Medical Association will assemble in convention—its twenty-second annual– at the great railroad centre, Fort Worth. Reduced rates over all the roads have been secured, delegates pay one and one-third fare going and return free. A ball and banquet will be given the delegates, and other features of social entertainment have been provided for. The indications are that there will be a very full attendance. The Association is rapidly growing in numbers and in influence, and the day will come when *not to be a member* of the State Association of Physicians will be a significant fact. It is to be hoped that the Committee of Arrangement will provide entertainment for the lady guests—the wives and daughters of members who will accompany them, and not let their exclusion from the banquet be as painfully conspicuous as it has heretofore been. It is expected that distinguished visitors from other States will be present, and we must "put the best foot foremost."

---

THE LONG TALKED OF BIOGRAPHY OF CONTEMPORARY PHYSICIANS OF TEXAS.—We state here in answer to the many inquiries, "when will the book be ready?" that the publication of the Biography of Contemporary Physicians of Texas so long in preparation by the editor of this JOURNAL, was abandoned as a separate publication, last fall, which fact was duly announced; and the data of such of the subscribers as gave their consent to the consolidation, was turned over to Dr. Tobin and Mr. L. E. Daniel, who had gotten up material for a work to be called "Types of Successful men" of Texas, to be incorporated in the latter work. The work is now nearly ready for delivering, the delay being in getting the engravings from New York. It will be a handsome book of some 700 pages in handsome morocco binding, and will contain biography and portrait of distinguished men of every class,—judges, lawyers, physicians, merchants, ranchmen, teachers, etc. All subscribers to the Contemporary Physicians of Texas who had paid subscription in part or in full will receive a copy, and their agreement with Dr. Daniel fully carried out. The work is being issued under his supervision, but his name will not appear in connection with it; in fact he has written most of the sketches and read most of the proof,—superintending the editing of the work. It will be ready within thirty days, without accident. Address J. J. Tobin or L. E. Daniel, Austin. Dr. Daniel has no interest in it.

---

AN UNFORTUNATE OCCURRENCE.—It appears from the dispatches received by the secular press to-day, April 11th, that while Dr. W. S. Carothers, (said to be late of Austin*) was alone with Dr. R. H. L. Bibb in the latter's office in Saltillo, Mexico, he was shot through the heart and instantly killed. Dr. Bibb declares it was entirely accidental; nevertheless he was arrested and charged with intentional killing, as there was said to be some feeling between them, and Bibb when excited, is known to have a violent temper. Dr. Carothers had returned to Saltillo to remove his furniture to Mexico City, to which point his wife had preceeded him. Whatever may be the facts, the JOURNAL deplores the occurrence and regrets that Dr..Bibb should have gotten into trouble.

[The Dr. Carothers referred to in the dispatches, it seems was Dr. E. J. Carothers, of San Antonio, a son of Dr. E. A. Carothers—a distinguished Texas surgeon—lately deceased, and who had resided many years in Mexico. Both father and son were members of the Texas State Medical Association. Dr. Bibb, we learn claims that Dr. C. accidentally shot himself. Dr. Bibb was to have been in Austin on the 20th on his way to the Medical Convention at Fort Worth on the 22nd.—ED.]

LATER.—Bibb released on bond.

* [We know of no such person who ever resided in Austin. There were two Drs. Carothers in San Antonio, A. E. and E. J.—ED.]

---

### ARRANGEMENTS.

FORT WORTH, April 11, 1890.

*Dr. F. E. Daniel, Editor Daniel's Texas Medical Journal:*

The hotels claim that they are always full and are not much inclined to give rates. The Pickwick and Ellis will charge $2.50 per day, and the Mansion, $2.00; The Grand, $2.00; Ginochio's, $2.50. The meetings will be held in the Opera House.

Yours, very truly, W. P. BURTS,

Chairman Arrangement Committee.

### Facts about Campho-Phenique.

### Campho-Phenique in Diseases of Women.

Dr. Walter B. Dorsett, Superintendent Female Hospital, St. Louis, says: As a local germicide, applied to the cervical canal in early cases of gonorrhœa in women, Campho-Phenique prevents endometritis and salphyngitis, and when used upon open chancroidal buboes, it promotes early granulation and healing.

Dr. P. P. Price, Professor Clinical Gynecology, Urinary and Rectal Diseases Electric Medical College, Cincinnati, writes: Having used Campho-Phenique with wonderful success in my practice for the past year in the treatment of rectal, vaginal and uterine diseases, I deem it but just to you that I give this expression of my gratitude for the great benefits derived from so valuable a remedy as Campho-Phenique. I regard it as a powerful antiseptic and local anæsthetic.

Dr. Walter Coles, late President St. Louis Obstetrical Society, writes: I am much pleased with Campho-Phenique. Its property of obtunding pain, in conjunction with its antiseptic and local alterative effects, renders it an admirable agent, when applied undiluted, in the treatment of erosions about the os uteri, and chronic ulcers and fissures of the anus. Mixed with vaseline it may be applied as a permanent dressing on absorbent cotton. Diluted with vaseline or lanoline it is an efficient and agreeable application in many skin diseases.

Dr. D. C. Knott, Argos, Ind., writes: Campho-Phenique is superior to any application I have ever used in the treatment of obstinate cases of ulceration of the os uteri; in fact, it is the only agent I have ever found that would cure such cases. Two cases were of two years standing and had resisted the most skillful treatment. Each was healed nicely with Campho Phenique, and the patients relieved of what had been a loathsome disease.

### Campho-Phenique in Surgical Practice.

Dr. W. H. Grayson, Surgeon to St. Mark's Railroad Hospital, Venice, Ills., says: I have used Campho-Phenique in all sorts of surgical procedures and believe it to be the best antiseptic known. I find it non-irritating, non-poisonous and highly germicidal. It corrects offensive odors and facilitates healing. In a word, Campho-Phenique is the only antiseptic agent I am acquainted with that possesses all the good qualities without any of the bad. It is the remedy par excellence in erysipelatous affections.

### Campho-Phenique in General Practice.

Dr. S. T. Yount, President Indiana State Medical Society, writes: I have given Campho-Phenique a careful and thorough trial in a great variety of cases, and find it better than any known remedy I have heretofore used. I have treated two cases of empyema with the most satisfactory results by the use of your remedy. I can cheerfully endorse it as the best antiseptic, germicide and disinfectant now in the market, and also can recommend it to the general practitioner as the very thing he needs for the various wounds, sores and injuries he may be called upon to treat.

### CIRCULAR.

OFFICE OF THE SURGEON GENERAL
OF THE UNITED CONFEDERATE VETERANS,
156 WASHINGTON AVE., NEW ORLEANS, April 9, 1890.

*To the Survivors of the Medical Corps of the Confederate States Army:*

COMRADES: The surrender of the Army of Northern Virginia, on this day twenty-five years ago, practically ended the struggle for the independence of the Southern States; and during this quarter of a century death has thinned our ranks, and our corps can now oppose but a broken line in the great struggle against human suffering, disease and death.

S. P. Moore, Surgeon General of the Confederate armies, is dead. Surgeons L. Guild, A. J. Foard, J. H. Berrien, J. T. Darby, W. A. Carrington, F. A. Ramsey, Samuel Choppin, R. J. Breckenridge, E. N. Coney; E. S. Gilliard, E. A. Flewellen, A. N. Tally, Paul F. Eve, O. F. Manson, Louis D. Ford, Habersham, James Bolton, and a host of other medical officers of the Confederate States Army are dead. The Association of the United Confederate Veterans was formed in New Orleans in 1888, the objects of which are historic, social and benevolent. Our illustrious Commanding General, John B. Gordon, Governor of Georgia, has ordered the United Confederate Veterans to assemble at Chattanooga on July 2nd, 1890. It is earnestly hoped that every surviving member of the medical corps of the Confederate Army, will meet with the United Confederate Veterans upon this important occasion, and promote by his presence and his counsels the sacred interests of the Association of the United Confederate Veterans.

It is of the greatest importance to the future historians, and also to the honor and welfare of the medical profession in the South, that careful records should be furnished the Surgeon General of the United Confederate Veterans, embracing the following data:

1. Name, age, nativity, date of commission in the Confederate States Army, nature and length of service of each and every member of the Medical Corps of the Confederate States Army.

2. Obituary notices and records of all deceased members of the Medical Corps of the Confederate Army.

3. The titles and copies of all field and hospital reports of the Medical Corps of the Confederate Army.

4. Titles and copies of all published and unpublished reports relating to military surgery and diseases of armies, camps, hospitals and prisons. The object proposed to be accomplished by the Surgeon General of the United Confederate Veterans is the collection, classification, preservation and final publication of all the documenss and facts bearing upon the history and labors of the Medical Corps of the Confederate States Army during the civil war, 1861–1865. Everything which relates to this critical period of our national history which shall illustrate the patriotic, self-sacrificing and scientific labors of the Medical Corps of the Confederate States Army, and which shall vindicate the truth of history, should be industriously collected, filed and finally published. It is believed that invaluable documents are scattered over the whole land in the hands of the survivors of the civil war of 1861–1865, which will form material for correct delineation of the medical history of the corps which played so important a part in the great historic drama. Death is daily thinning our ranks, whilst time is laying its heavy hands upon the heads of those whose hair is already whitening with the advance of years and the burden of care.

No delay, fellow-comrades, should be suffered in the collection and preservation of these precious documents. This task of collection of all documents, cases, facts relating to the medical history of the Confederate Army invites the immediate attention and co-operation of the honored comrades and beloved compatriots throughout the South.

Respectfully, your obedient servant,

JOSEPH JONES, M. D.,
Surgeon-General U. C. V.

[We publish the above with pleasure at the request of Dr. J. Jones, the Surgeon-General.—ED.]

